# Prevalence of Oral Human Papilloma virus infection in an East African HIV/AIDS cohort: A cross-sectional study

**DOI:** 10.21203/rs.3.rs-5594024/v1

**Published:** 2025-04-21

**Authors:** Ian G. Munabi, Adriane. Kamulegeya, Dunstan. Kalanzi, David P. Kateete, Fred Collins. Semitala, Catherine- Lutalo. Mwesigwa, Samuel. Kalungi, Jennifer E. Cameron, Kimon. Divaris, William. Buwembo

**Affiliations:** Department of Anatomy, School of Biomedical Sciences, Makerere University College of Health Sciences, Kampala; School of Dentistry, Makerere University College of Health Sciences, Kampala; School of Dentistry, Makerere University College of Health Sciences, Kampala; Department of Immunology, School of Biomedical Sciences, Makerere University College of Health Sciences, Kampala; Department of Medicine, School of Medicine, Makerere University College of Health Sciences, Kampala; School of Dentistry, Makerere University College of Health Sciences, Kampala; Department of Pathology, Mulago National Referral and Teaching Hospital, Kampala; Louisiana State University Health Sciences Center: New Orleans, LA; Adams School of Dentistry, University of North Carolina at Chapel Hill; Department of Anatomy, School of Biomedical Sciences, Makerere University College of Health Sciences, Kampala

**Keywords:** Oral HPV, People Living with HIV, Multiple infections

## Abstract

**Background:**

Little is known about the prevalence of Oral Human Papilloma Virus (OHPV) in people living with HIV (PLWHIV) in the East African region. The objective of this study was to document the prevalence and types OHPV infection in a large cohort of PLWHIV attending an urban HIV clinic in Eastern Africa

**Methods:**

This was a cross-sectional study among 1,715 participants of the Makerere University Joint AIDS Program clinic, located in Kampala, Uganda, in East Africa. A salivary sample was collected from which DNA was extracted and subjected to a Polymerase Chain Reaction (PCR) based typing. Data analysis was carried out among participants with complete data (n = 1,243) and relied on descriptive statistics (frequencies, percentages), bivariate testing and multivariate regression modeling, using a conventional p < 0.05 statistical significance threshold.

**Results:**

Participants had a mean age of 45 (SD = 10) years, most (69%) were female, and 67% were HPV positive. There was no association between participants’ age or gender and the likelihood of them being diagnosed with HPV infection in this study. HPV type 45 was the most frequently (16%) identified HPV, while HPV type 18 (5%) and HPV type 16 (2.3%) were less frequently identified. Most of the HPV positive samples had more than one HPV type detected.

**Conclusion:**

This study highlights the high prevalence of OHPV among PLWHIV, with HPV type 45 being the most frequently detected type, and smaller frequencies of vaccine targeted HPV 16 and HPV 18. Our findings highlight the need for continued surveillance and typing of circulating OHPV strains, particularly among PLWHIV, to inform evidence-based preventive strategies.

## Introduction

Oral human papilloma virus (OHPV), has been established as a major contributor to oropharyngeal cancers, especially among young people [1]. OHPV infections are usually self-limiting due to eventual clearance by the host’s immunity in most healthy individuals [2–4]. The ongoing global Human Papilloma Virus vaccination efforts may over time modify the epidemiology of OHPV related cancers, although some high-risk genotypes such as type 45 are not included in the vaccine [5], cross reactivity from existing vaccines has been reported to cover both 31 and 45 [6, 7]. This cross reactivity has been reported among people living with HIV as well [6, 7]. Vaccine-related population-level modification of the prevalent oral HPV types has been observed consistently in different parts of the world [8, 9]. Even with this vaccine-related HPV prevalence modification, there remains a strong need for continued surveillance and typing of the circulating OHPVs within our populations for evidence-based preventive planning [8]. This is important because of the finding of the most recent review [6] which shows that: Cross-protection antibody response to non –vaccine HPV types varies widely, Antibody response is mostly detected for HPV 31 six months after third vaccine dose, Cross-protection antibody response is lower than that with direct protection and Data on duration of immune response to non –vaccine HPV beyond two years is limited.

PLWHIV, especially those with multiple sexual partners, have a higher risk of exposure to the OHPV infections compared to their HIV negative counterparts [4, 10–13]. In PLWHIV, low CD4 cell counts and high HIV viremia are reportedly strongly associated with the presence of HPV [13, 14]. This may be due to the way the HIV infection modifies the body’s natural viral clearing process leading to persistent OHPV infections [2, 15]. This has been demonstrated in modeling studies that have shown that immune suppression, as manifested by low CD4 cell count, plays a major role in the non-clearance of oral HPV in people living with HIV [16]. Similar conclusions have been drawn from studies on people living with HIV that examined the prevalence of HPV across different anatomical sites of the body [17]. In addition, some Anti-retroviral (ART) drugs like the protease inhibitors have been shown to increase access to the basement membrane on initiation of treatment [18]. These observations may in part explain the high prevalence of OHPV [13], and/or its persistence of up to seven years [15], in PLWHIV. Except for a recent study in a south African HIV positive population that reported a low prevalence of OHPV [19], there is paucity of data regarding the prevalence of OHPV in PLWHIV from the East African region. The objective of the work reported in this manuscript was to document the prevalence and types of oral HPV in a cohort of people living with HIV attending an urban HIV clinic in Eastern Africa.

## Methods

**Figure 1:**
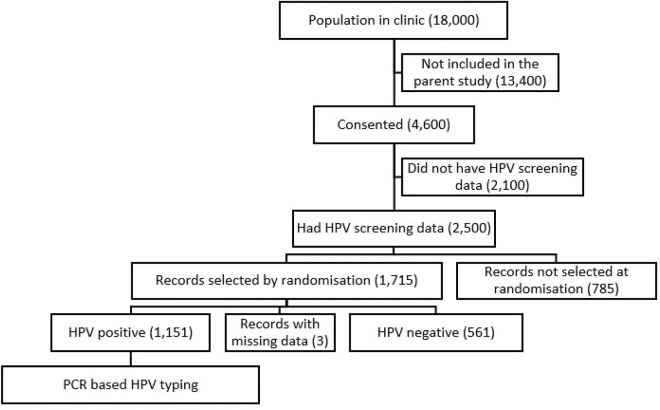
Participant flow diagram

In this cross-sectional study, participants were drawn from Makerere University Joint AIDS Program clinic (MJAP), situated in Kampala Uganda which serves a total of 16500 people living with HIV drawn from both the urban and peri-urban populations of south-central part of Uganda in East Africa [20]. Most of the PLWHIV at this clinic are virologically suppressed on their respective HAART regimens [21] The data in this report were generated from a sub-sample of the parent case control study that examined OHPV, microbiota and cancer in PLWHIV [22]. In the parent study, participants who were registered patients in the clinic, above the age of 18-years and had provided informed consent, were consecutively recruited over a 6-month period to attain a target sample size of 4,600. At the time of the conduct of this study, 2500 of these participants’ samples had undergone HPV DNA screening and met inclusion criteria for inclusion in this study as shown in the participant flow diagram (see figure 1). A target sample size of 1,715 participants was drawn from the above records of the primary study with HPV test results. This was calculated using the online sample size calculator [23], based on the following assumptions: among a total 2,500 records that had been screened, hypothesized frequency of participants with oral HPV in the study population of 50%, absolute confidence limits 2% [24], design effect of 1 and power of 95%, that resulted in an estimated sample size of 1,225 participant records. To this we added a 40% allowance for errors and missing information resulting in a final sample size of 1,715 [25].

### Study procedure:

Participants were recruited as they presented to the clinic for their routine 6-monthly HIV-related drug refills. During the clinic visit, potential participants were approached and asked to participate in the study. After consenting, each participant was asked to provide a sample of saliva for five minutes. After five minutes, participants were asked to top up their samples to 5mls. These saliva samples were immediately placed on ice for transfer to the laboratory within two hours of collection. In the lab, each sample was aliquoted into two 2ml batches that were both centrifuged at 4000 revolutions per minute for four minutes. The supernatant from each of these samples was discarded and one of the remaining pellets was immediately placed in Cell lysis solution of the DNA extraction Kit (D4069, Zymoresearch, CA, USA). The second pellet was placed in a cryovial at minus 20 degrees Celsius for later transfer to minus 80 degrees Celsius for long term storage. The remaining 1ml sample was stored immediately at minus 20 degrees Celsius and later to minus 80 degrees for long term storage. Other data that were extracted from participant records included age, sex and marital status, and were captured using a study questionnaire or abstracted from the clinic records using a study clinical history data abstraction form that was completed by one of the clinics nursing staff were collected and managed using REDCap electronic data capture tools hosted at Makerere University [26].

### DNA Extraction:

DNA was extracted using Quick-DNA Mini prep Plus Kit (D4069, Zymoresearch, CA, USA), with overnight protein kinase digestion, as per the manufacturer’s instructions. After extraction the DNA from each sample was quantified using a Nanodrop colorimeter (Thermofisher scientific, MA, USA) following the manufacturer’s instructions.

### HPV screening:

Following extraction, as described above, 10-micro-liters of each sample were subjected to screening using FAP 59 (5’TAACWGTIGGICAYCCWTATT3’) and FAP 64 (5’CCWATATCWVHCATITCICCATC3’) primers that target a conserved region within the L1 open reading frame (ORF) of the HPV genome [12]. DreamTaq DNA polymerase were used in this assay condition: 5 min at 94 °C, 40 cycles (denaturation 94 °C/30 s, annealing 52 °C/45 s, and extension 72 °C 1 min) followed by a final extension step at 72 °C for 7 min. The 490bp band HPV positive PCR products were visualized using 2% agarose gel with ethidium bromide. Samples without any band from the FAP primers were subjected to a confirmatory pcr screening for 150bp bands using the double nested PGmy9/11 and Gp+5/+6 primers and protocol [27]. The PCR positive and negative controls were sequenced OHPV DNA and distilled water respectively.

### Typing HPV PCR

All the HPV positive samples in the study pool were subjected to further PCR based typing using the previously described Sotlar method [28], with the following modifications. HPV DNA was amplified using a single consensus forward primer, GP-E6–3 F [GGGWG KKACT GAAAT CGGT], and two consensus reverse primers GP-E7–5B [CTGAG CTGTC ARNTA ATTGC TCA] and GP-E7–6B – [TCCTC TGAGT YGYCTAATTG CTC]. These primers give a 602bp to 666bp PCR product from the E6 and E7 regions of the HPV genome, that were visualized using 2% agarose gel containing ethidium bromide using the ebox machine (Vilber E-box, Vilber, Deutschland GmbH. Wielandstrasse 2, Germany). Then four second round primer-cocktail-sets, were used for the genotyping [28]. Both first and second round PCRs were performed in a final volume of 50 μL and each PCR mixture contained 9 mM Tris-HCl (pH 9.0), 2.0 mM MgCl2, 0.2 mM of each dNTP, 320 nM of each of the primers and 1.25 U of DreamTaq polymerase. The amplifications were carried out using a thermal cycler a Simply Amp Thermocycler (Applied Biosystems, Waltham, MA, USA) following the above modified Sotlar method parameters [28]. The typing was aided by the ebox analyse software (Vilber E-box, Vilber, Deutschland GmbH. Wielandstrasse 2, Germany), that was used to measure the band base pair weights in reference to a 100bp ladder using the previously published reference levels to identify the oncogenic (Hpv_16, Hpv_18, Hpv_31, Hpv_33, Hpv_35, Hpv_39, Hpv_45, Hpv_51, Hpv_52, Hpv_56, Hpv_58, Hpv_59, Hpv_66, Hpv_68), and non-oncogenic (Hpv_11/6, Hpv_42, Hpv_43, Hpv_44) types of HPV [28]. Typing data were captured in an excel sheet as either present or absent for each of the four primer cocktail sets per sample as described [28].

### Data analysis

All data were saved in Microsoft excel (Microsoft 365 version 2306) and imported into R statistical computing environment version 4.4.1 for cleaning, correction of format or range errors before eventual analysis. The results were reported initially using descriptive statistics including frequencies and percentages. Prevalence ratios (PR) were obtained by exponentiating beta coefficients and summarized in tables using the broom.mixed [29], and flextable [30] R packages. For these count data, models were created using the Lme4 [31], and glmmTMB [32]. Additional modeling for possible HPV vaccination effect was done to obtain the PR of having an infection due to HPV 16 and HPV 18 compared to the other HPVs with respect to one’s age for the 25-year-olds and above. Additional modeling for possible HPV vaccination effect was done with Lme4 [31] to obtain the odds of having an infection due to HPV 16 and HPV 18 compared to the other HPVs with respect to the categorized age and gender. All explanatory variables in the regression models were tested for multicollinearity using the variance inflation factor (VIF) related functions in the car (3.1–3) and performance (0.13.0) R packages. A VIF value of greater than 10 was considered as indicative of multicollinearity or suggestive of strongly correlated variables. Only complete records were included in the data analysis and the level of significance was set at 0.05 for all statistical tests.

### Ethical considerations

Written informed consent was obtained from all respondents. Maximum confidentiality and privacy were maintained at all levels of data and sample collection and processing. This was in addition to presenting the study protocol to the Makerere University School of Medicine Research and Ethics Committee (Mak-SOMREC-2–22-451) and the Uganda National Council of Science and Technology (HS241ES) a national Research regulatory body prior to the initiation of data collection.

## Results

As shown in the study flow diagram (see Fig. 1), of the 4600 participants recruited into the parent study at the time of analysis, 2,500 had completed the HPV screening. A total of 1,715 (68.6%) records were randomly selected from this pool using computer-generated random numbers for inclusion in analysis reported in this manuscript.

Table 1 provides a summary of the descriptive statistics of the selected participants with an average age of 45years (SD = 10). Most of the participants were female and HPV positive. There was no association between participants’ age or gender and the likelihood of being diagnosed with HPV infection in this study. There were also no significant differences in HPV positivity with respect to level of education, religion, or marital status. The VIF values for the explanatory variables were between 1.4 to 1.68 for this regression model (see supplementary data).

Table 2 provides a summary of the HPV types detected from participants’ saliva samples. In this table 2, HPV45 was the most frequently identified (16%) HPV type. In this table, it is only the HPV types 11 or 6, 42, 43 and 44 that represent the non-oncogenic types of HPV that were detectable using the study typing method. There were a total of 550/1980 (28%) HPV infections detected and attributed to these non-oncogenic HPV viruses. The remaining detected infections (72%) were due to oncogenic HPV viruses. In table 2, note that HPV 45 (16%) was the most detected oncogenic and overall type of HPV in this population. HPV 16 (2.3%) and HPV 18 (5%), though present, accounted for a smaller proportion of oncogenic HPV virus infections. There was no significant difference in the odds of a sample having either HPV 16 or HPV18 compared to the other HPV types with respect to the participants’ age (adjusted odds ratio controlling for gender = 0.91, 95% CI 0.36 to 2.30, p-value = 0.85) or gender (adjusted odds ratio controlling for age males = 1.11, 95% CI 0.78 to 1.59, p-value = 0.56). It is important to note that some participants had multiple infections, as evidenced by the detection of more than one type of HPV in a sample, including combinations of both the oncogenic and non-oncogenic types of HPV, as reported in Table 1.

## Discussion

We set out to determine the prevalence and types of oral HPV in a cohort of people living with HIV attending an urban HIV clinic in Eastern Africa. We found that 67% of our participants’ samples were HPV positive. The high prevalence of OHPV in our study population may be attributed to the immunocompromised status of PLWHIV, which impairs their ability to clear the virus [2, 13]. Our findings indicate a high prevalence of OHPV among PLWHIV, with HPV type 45 being the most frequently detected. The detection of HPV type 45 as the most frequently identified type in our study is noteworthy. HPV 45 is a high-risk genotype that is not covered by the current HPV vaccines [5]. It is important to note that HPV 45 is the third most carcinogenic type of HPV after both HPV 16 and HPV 18 [33]. HPV 45 is also in the same phylogenetic species group (alpha-7) as HPV 18 [34]. The fact that it is in the same phylogenetic group as 18 may partly explain the presence of cross reactivity when people are vaccinated with either bi-valent or quadra-valent HPV vaccines [6]. HPV 18 infections are characterized by integration into the host’s DNA, one of the first steps towards converting host cells into cancer cells, thus we should expect HPV 45 to have the same behavior. The observed prevalence of OHPV in our study is consistent with previous reports from other parts of the world, which have shown that PLWHIV are at an increased risk of OHPV infections compared to the general population [4, 8–11].

Our findings indicate that the majority of OHPV infections in our study population were multiple infections, comprising both oncogenic and non-oncogenic types of HPV. This is consistent with previous reports, which have shown that multiple HPV infections are common among PLWHIV [11, 12]. In this study the other viruses that together with HPV 45, accounted for 40% of the infections in study participants, were HPV 66, HPV 58 and HPV 31. HPV 66 belongs to the alpha-6 group, while both HPV 58 and HPV 31 belong to the alpha-9 group that also includes HPV 16 [35]. The relatedness of these HPVs to the oncogenic HPV 16 and HPV 18 is emphasized here to demonstrate their high risk for integration that leads to persistence of infection and eventual oncogenicity [36]. In the presence of mixed high-risk HPV co-infections as was seen in this study the risk of oncogenicity may increase substantially [37]. This further highlights the need for continued surveillance and typing of circulating OHPV strains in our population, particularly among PLWHIV, for evidence-based preventive strategies [38]. Despite the fact that both cross reactivity for 31 and 45 have been reported when the present HPV vaccines are used [7, 39], it is noteworthy to factor in the completion rate of HPV vaccination that stands at only 43.3% in Uganda [40]. This may point to insufficient protection for our population and less likelihood of attaining herd immunity hence the need to continuously carry out community surveillance [41].

In this study we found a few participants samples containing HPV 16 (2.5%) and HPV 18 (5%). High prevalence of HPV 16 has been associated with oral sexual practices; e.g., in the United Kingdom, young healthy adults were found to have a 19% prevalence of HPV 16 that was attributed to oral sexual practices [42]. In comparison, HPV 16 was the most frequently identified type of HPV affecting 1% of the healthy U.S. general population [43]. It has been reported that the prevalence of HPV in people with HIV is 2–3 times higher than that in the general population [44]. This suggests that our finding of a 2.5% prevalence for HPV 16, while low, is within expectation. Since 2014, these two HPV types have been the target of a nationwide vaccination campaign in Uganda, using routine immunizations to eliminate cancer of the cervix by targeting girls under the ages of 9–14 years [40]. Despite the concerted efforts on the part of government, it is reported that the uptake of this vaccine has remained low. Also, our study participants were on average 45 years of age thus outside this age group at the time of the study recruitment. Despite this, in sub-analyses, we found there was no difference in the odds of a sample having either HPV 16 or HPV18 compared to the other HPV types with respect to participants’ age or gender. This may explain why we were able to detect these two types of HPV virus in samples from our study population.

The strengths of our study include the large sample size and the use of a robust methodology for HPV detection and typing. However, our study had some limitations. First, the cross-sectional design of our study precludes any causal inferences between OHPV infections and HIV-related factors. Second, our study population was limited to PLWHIV attending an urban clinic, which may not be representative of the broader population of PLWHIV in Eastern Africa. There were also possible biases in sample collection or errors in molecular diagnosis as confirmatory sequencing was not done. For this study we assume that the participants may not have received any of the HPV vaccines, even though they have been included in the routine national vaccination program for girls under the age of 9–14 years since 2015 [40]. Despite these limitations the study findings provide a basis for initial inferences into the prevalence and nature of OHPV infections affecting this vulnerable population at this site to inform future studies.

## Conclusions

Our study highlights the high prevalence of OHPV among PLWHIV in Eastern Africa, with HPV type 45 being the most frequently detected type. We also detected the vaccine-targeted HPV 16 and HPV 18 types at lower frequencies, despite an ongoing national HPV vaccination campaign for about 10 years. Our findings highlight the need for continued surveillance and typing of circulating OHPV strains, particularly among PLWHIV, to inform evidence-based preventive strategies.

## Figures and Tables

**Table 1:** Participant Characteristics

Characteristic	N = 1,712^1^
Age	45 (10)
Gender	
Male	534 (31%)
Female	1,178 (69%)
Marital Status	
Single	112 (6.5%)
Married	700 (41%)
Divorced/separated	540 (32%)
Cohabiting	140 (8.2%)
Widowed	220 (13%)
Religion	
Catholic	567 (33%)
Protestant/Anglican	464 (27%)
Pentecostal/Born Again	318 (19%)
Muslim	320 (19%)
Adventist	36 (2.1%)
Atheist	2 (0.1%)
Traditionalist	3 (0.2%)
Other	2 (0.1%)
Level of Education	
Postgraduate Degree	3 (0.2%)
College/University completed	73 (4.3%)
A level completed/ Tertiary institution after O level (S6)	145 (8.5%)
Secondary school (O level) completed (S4-S5)	265 (15%)
Primary school completed (P7-S3)	566 (33%)
Less than primary school (P1-P6)	548 (32%)
No formal schooling (No Education)	112 (6.5%)
How often did you go to the dentist during the past 12 months?	
Once	221 (13%)
Twice	94 (5.5%)
Three times	20 (1.2%)
Four times	5 (0.3%)
More than four times	16 (0.9%)
I had no visit to dentist during the past 12 months	1,121 (65%)
I have never received dental care/visited a dentist	232 (14%)
I don’t know/don’t remember	3 (0.2%)
Salivary nowrate per minute	1.20 (0.59)
Number of HPV types detected	
negative	561 (33%)
No_reaction	478 (28%)
Single	199 (12%)
Multiple	474 (28%)
Result of HPV testing	
negative	561 (33%)
positive	1,151 (67%)
Cigarette consumption	
Never (1)	1,634 (95%)
Seldom (2)	15 (0.9%)
Several times a month (3)	4 (0.2%)
Once a week (4)	6 (0.4%)
Several times a week (5)	27 (1.6%)
Every day (6)	26 (1.5%)
Alcohol consumption	
Less than 1 drink	5 (0.3%)
1 drink	118 (6.9%)
2 drinks	136 (7.9%)
3 drinks	85 (5.0%)
4 drinks	40 (2.3%)
5 or more drinks	127 (7.4%)
Did not drink alcohol during the past 30 days	1,201 (70%)

1 Mean (SD); n (%)

**Table 2.** HPV types

HPV	Number (col %)
Hpv_11	11 (0.6%)
Hpv_16	45 (2.3%)
Hpv_18	98 (5.0%)
Hpv_31	135 (6.9%)
Hpv_33	78 (4.0%)
Hpv_35	47 (2.4%)
Hpv_39	62 (3.2%)
Hpv_42	289 (15%)
Hpv_43	207 (11%)
Hpv_44	57 (2.9%)
Hpv_45	306 (16%)
Hpv_51	70 (3.6%)
Hpv_52	65 (3.3%)
Hpv_56	74 (3.8%)
Hpv_58	78 (4.0%)
Hpv_59	49 (2.5%)
Hpv_66	148 (7.6%)
Hpv_68	131 (6.7%)
Total	1,950 (100%)

## Data Availability

Data is provided with in the manuscript

## List of abbreviations

HIV: Human immunodeficiency virus
OHPV: Oral Human Papilloma Virus
PLHIV: People living with HIV
HAART: Highly Active Antiretroviral Therapy
HPV PCR: Human Papilloma Virus Polymerase Chain Reaction
ARTs: Antiretroviral therapy’s
MJAP: Makerere Joint Aids Program
SOMREC: Makerere University School of Medicine Research and Ethics committee
